# Cryosurgery and vascularized fibular graft reconstruction in proximal tibia osteosarcoma in young children: A case report^☆^

**DOI:** 10.1016/j.ijscr.2021.106568

**Published:** 2021-11-03

**Authors:** Uno Surgery Erwin, Sigit Daru Cahyadi

**Affiliations:** a Department of Orthopaedic and Traumatology, Faculty of Medicine Universitas Indonesia, Dr. Cipto Mangunkusumo Hospital, Indonesia; b Department of Orthopaedic and Traumatology, Faculty of Medicine Universitas Indonesia, Persahabatan General Hospital, Indonesia

**Keywords:** Osteosarcoma, Very young children, Cryosurgery, Vascularized fibular graft

## Abstract

**Introduction:**

Osteosarcoma epidemiology in children younger than 5-year-old is very rare. Currently, limb salvage surgery becomes the preferred treatment in osteosarcoma. Wide excision using cryosurgery has been reported as an effective and safe procedure for malignant bone tumors.

**Case report:**

A 5-year-old girl was presented with pain and a lump on her right knee. Physical examination showed a solid circumferential mass in the proximal tibia, with limited range of motion due to mass and pain. Osteoblastic lesion with a discrete margin and narrow transition zone on the proximal tibia from plain radiograph and magnetic resonance imaging (MRI) examination. Histopathological examination suggested osteosarcoma with a giant cell rich osteosarcoma subtype. Three cycles of neoadjuvant chemotherapy was conducted with cisplatin, ifosfamide, and adriamycin. We performed limb salvage surgery by wide excision with cryosurgery and vascularized fibular graft reconstruction. Wide excision was performed with the respect to preserve the epiphyseal plate. The proximal tibia segment was recycled using liquid nitrogen and re-implanted, fixed with a locking 2.7 mm T-plate and a straight reconstruction 2.7 mm reconstruction plate. Vascularized fibular graft was used to fill the bone defect on the medial side. Post-operative radiograph showed the plate and screws are well-fixated and the post-operative histopathological examination confirmed the diagnosis of conventional osteosarcoma HUVOS I. There was no post-operative complication observed, and the functional outcome was good.

**Conclusions:**

Cryosurgery and vascularized fibular graft is a viable reconstructive option for proximal tibia osteosarcoma in very young children.

## Highlights

• Osteosarcoma in a very young child is rare.
• Few cases of osteosarcoma in very young child treated with cryosurgery
• Wide excision with cryosurgery and vascularized fibular graft reconstruction showed excellent functional outcome.

## 1 Introduction

Osteosarcoma is one of the most common bone malignancies, only second to multiple myeloma in incidence [1]. Osteosarcoma has a bimodal distribution with the highest incidence in the adolescent and geriatric population. Osteosarcoma epidemiology in children younger than 5-year-old is very rare. A previous study reported the prevalence of 1% of all patients with high-grade osteosarcoma [2]. The difference in presentation, treatment, and prognosis of osteosarcoma in young children was reported in the previous study with a higher proportion of upper limb for the primary site, higher rate of metastasis, higher rate of amputation, and lower overall 5-year survival compared to the adolescent population [3].

Currently, the management of osteosarcoma consists of chemotherapy, surgical resection, and radiation therapy [4]. However, these treatment approaches have not been well studied in young children [3]. Limb salvage surgery has been considered as the preferred treatment in osteosarcoma with improved 5-year survival, limb function, and quality of life [5], [6]. Previous study has shown limb salvage surgery with endoprosthetic devices, rotationplasty, extracorporeal irradiation, and autologous bone graft as reconstruction options in the pediatric population [7]. Cryosurgery has also been reported as an effective and safe procedure for malignant bone tumors [8]. We reported a case of a five-year-old child with proximal tibia osteosarcoma treated with wide excision with cryosurgery and vascularized fibular graft reconstruction. This case report has been reported in line with the SCARE Criteria [9].

## 2 Case illustration

A 5-year-old girl was presented with a lump and pain in her right knee. The patient was with a history of falling from a bike two months prior to the admission with no significant injury related to the fall. General examination was within normal limit. The patient complains of unexplain loss of weight. Pain was felt out of proportion and worsen at night.

**Fig. 1:**
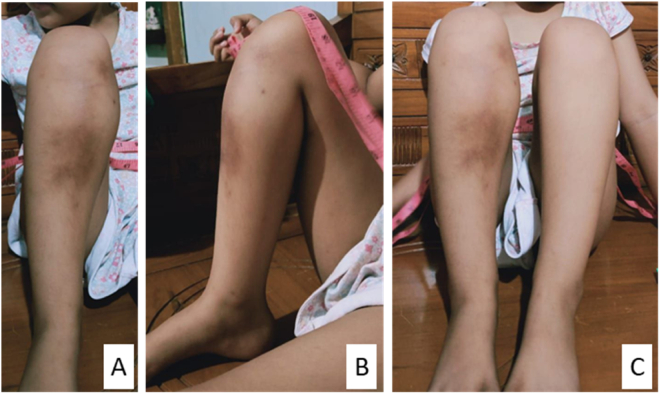
Physical examination showing a circumferential mass of the proximal right tibia. A) Anteroposterior view; B) lateral view; C) comparison between right and left cruris.

**Fig. 2:**
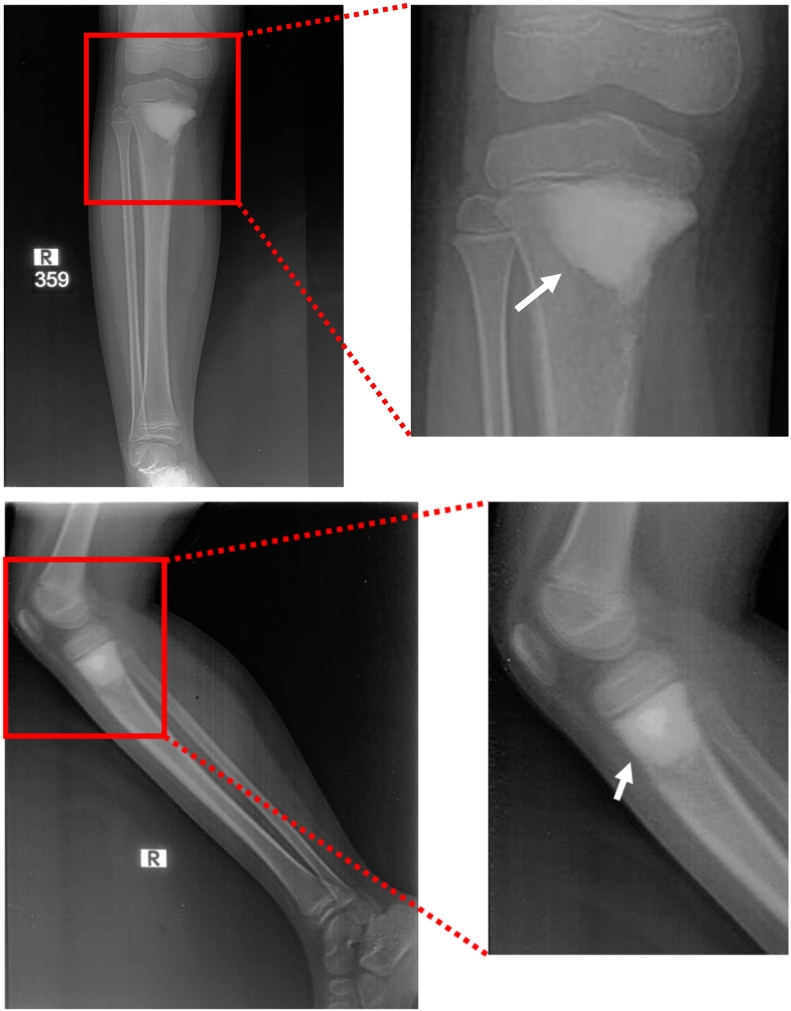
X-ray examination of the cruris showing osteoblastic lesion (white arrow) of the proximal tibia in anteroposterior and lateral view.

**Fig. 3:**
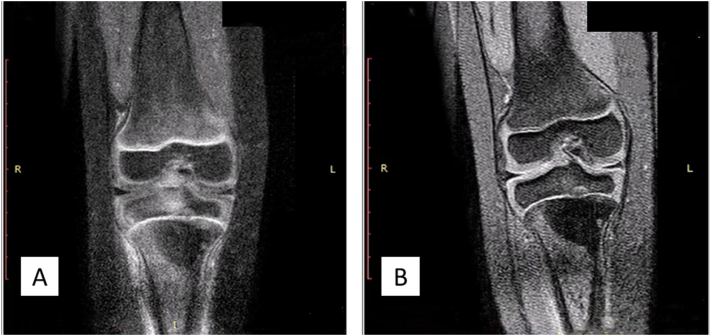
MRI examination of the right proximal tibia. A) T1 coronal MRI examination; B) T2 coronal MRI examination.

**Fig. 4:**
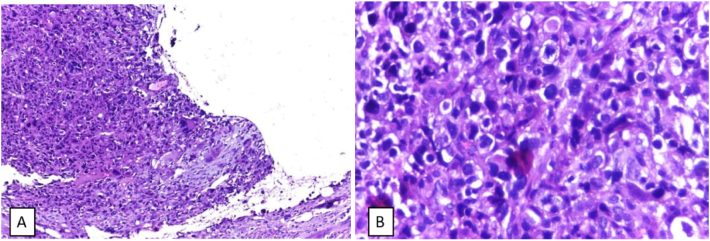
Histopathological examination. A) Multinucleated giant cell; B) pleomorphic hyperchromatic tumor cells with eosinophilic matrix.

Physical examination of the right knee showed a solid circumferential mass (diameter 30 cm, contralateral 26 cm) on the proximal tibia (Fig. 1). The range of motion of the right knee is limited to 120° compared to 140° in the contralateral side due to the mass and pain. A plain radiograph was obtained which showed an osteoblastic lesion on the proximal tibia with a discrete margin and narrow transition zone. The cortex of the bone was intact with no periosteal reaction (Fig. 2). The margin of the lesion was further described on magnetic resonance imaging (MRI) with contrast (Fig. 3). A computed tomography scan of the chest showed no metastatic nodules. Histopathological and immunochemistry examination confirmed the diagnosis of osteosarcoma with a high degree giant cell-rich subtype (Fig. 4).

Three cycles of neoadjuvant therapy regiment with cisplatin, ifosfamide, and adriamycin were administered to the patient. Multidisciplinary approach surgery with plastic surgeon and cardio thoracic vascular surgeon was conducted in this patient.

**Fig. 5:**
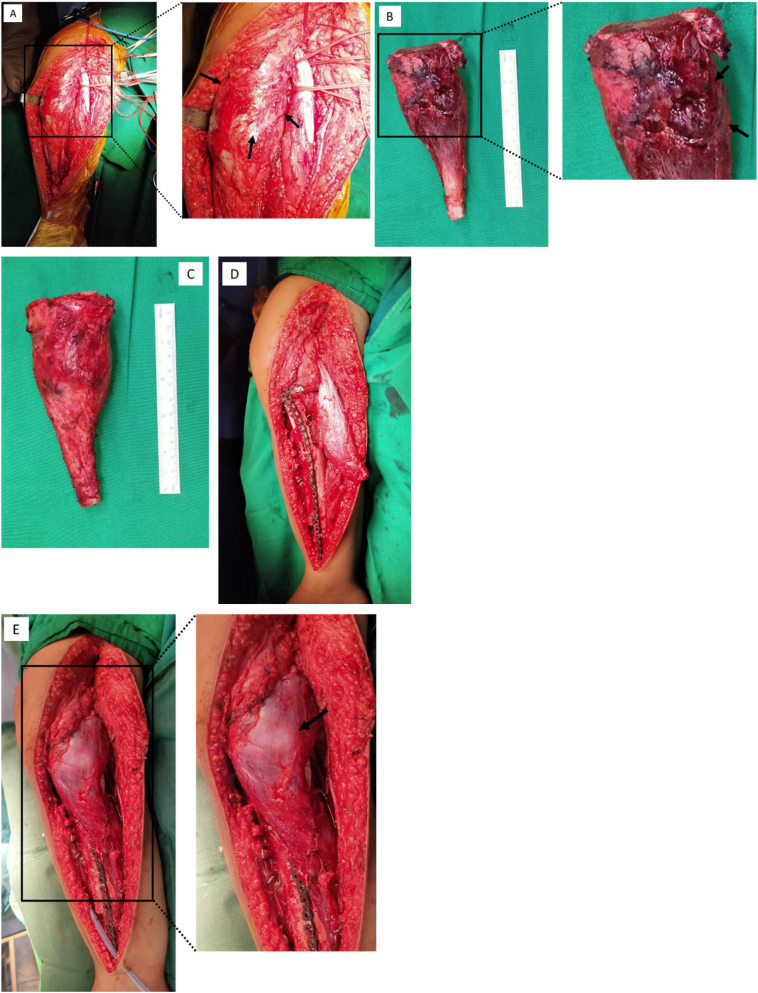
Intraoperative: A) Tumor exposed (arrow); B) gross pathology (arrow: tumor margin); C) after cryotherapy; D) construction after fixation; E) final construct after reconstruction (arrow: hemigastrocnemius flap).

The surgery was performed with a torniquet in a supine position with an anteromedial approach on the proximal tibia. The popliteal bundle was then identified and preserved. Ligation and anastomosis of the vein involved with the tumor were performed prior to wide excision. Wide excision was performed with the respect to preserve the epiphyseal plate proximally and 13 cm of the epiphyseal plate distally. The proximal tibia segment was clear from the tumor with osteotome and bone curette (Fig. 5)

**Fig. 6:**
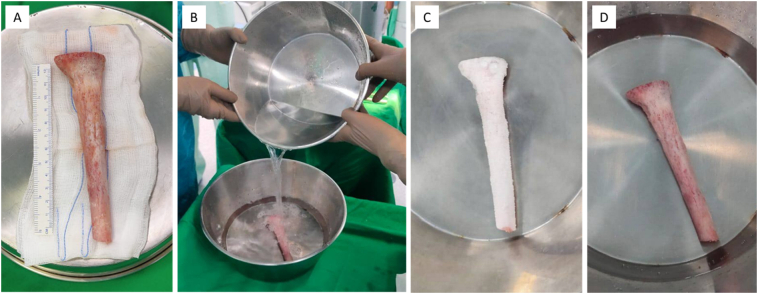
Cryosurgery process: A) Proximal tibia segment preparation; B) liquid nitrogen pouring; C) recycling process in liquid nitrogen for 40 min; D) thawing in room temperature for 15 min.

The proximal tibia segment was recycled using liquid nitrogen for 40 min. The recycled autograft was left to be thawed at room temperature for 15 min and soaked in 160 mg gentamycin saline for 5 min (Fig. 6). The recycled autograft was re-implanted and fixed with a locking 2.7 mm T-plate and a straight reconstruction 2.7 mm reconstruction plate (Heng Jie, China). The bone defect on the medial side was filled with a vascularized fibular graft which was harvested by prior to wide excision. The remaining bone defect was filled with bone graft and bone substitutes. A medial hemigastrocnemius flap was used for soft tissue coverage.

**Fig. 7:**
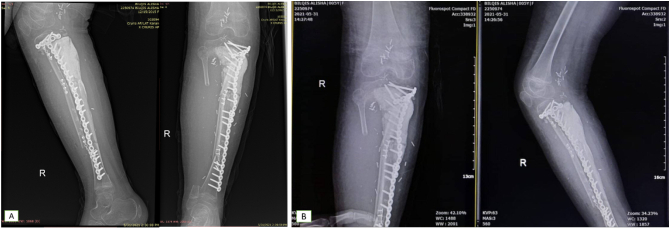
Post-operative X-ray of the: A) Cruris; B) knee.

Postoperatively we performed an X-ray to evaluate the surgery. From the X-ray, the plate and screws are well-fixated (Fig. 7). Postoperative histopathological examination confirmed the diagnosis of conventional osteosarcoma HUVOS 1 with free tumor margin.

Postoperative condition showed good surgical and functional outcomes. Postoperative complications did not occur in this patient. Radiological union was achieved in 1 year followup with the ROM of 120° in the affected knee. Functional outcome was calculated with musculoskeletal society tumor score (MSTS) with the result of 76,6%.

## 3 Discussion

We presented the case of osteosarcoma in 5-year-old children treated with cryosurgery. Cryosurgery is the therapeutic use of cold to cause tissue necrosis with the goal of ablating the tissue. Because bone necrosis occurs at temperatures below −21 °C, cryosurgery with liquid nitrogen is beneficial in the treatment of bone malignancies. The major processes of liquid nitrogen induced cellular necrosis are thought to be the production of intracellular ice crystals and membrane rupture [10]. The use of intralesional excision followed by cryosurgery has been reported as effective as wide lesion in benign, aggressive, and metastatic lesion [11]. Previous report has shown successful results with cryosurgery in several muskuloskeletal tumors such as giant cell tumor, chondrosarcoma, enchondroma, aneurysmal bone cyst, fibrous dysplasia, and others [10], [12], [13]. The use of cryosurgery in primary bone sarcoma such as osteosarcoma, chondrosarcooma, and other has been described in previously with good clinical outcomes [8], [10], [14].

In this case, we performed a limb salvage surgery with adjuvant cryosurgery and vascularized fibular graft followed by hemigastrocnemius muscle flap. Limb salvage surgery is defined as a surgical procedure to preserve or restore the function of bone and joint after resection of a malignancy [15], [23]. Limb salvage surgery could only be done if the malignancy is sensitive to chemotherapy, a surgical margin of wide excision could be achieved, good overall physical condition and preservation of the main neurovascular structure could be obtained [15], [24]. The advantage of limb salvage surgery has been shown in previous studies [5], [6].

The use of liquid nitrogen as cryosurgery for osteosarcoma has been reported in a previous study [14], [16], [17]. However, the use of cryosurgery as a reconstructive method of osteosarcoma in very young children has not been reported in a previous study. Cryosurgery has been described by applying the liquid nitrogen into the tumor cavity or soaking the resected segment with liquid nitrogen [16], [18]. In this case, curettage and drilling to the bone bed and wall were performed to maximize the contact between the liquid nitrogen and the most inner layer of the bone [8], [18]. Adequate contact of the tumor margin is important to ensure the delivery of extreme temperature that destroy the malignant cell [19], [25]. Previous study has shown that inadequate freezing may predispose to local recurrence of bone tumor [20], [22]. The duration of freezing in the liquid nitrogen, in this case, is longer than in the previous study, with 40 min of freezing compared to 20 min [16]. The duration of 40 min in cryosurgery has not been described in previous study. The extended duration of freezing was considered due to suboptimal contact between the bone and the inner layer of the bone. The use of bone graft or bone cement as a mechanical support to the recycled autograft has also been reported in the previous study [21], [26]. The efficacy of cryosurgery has been reported in 10 patients with no local or systemic recurrence in all subjects and a functional score of 82.4% [16]. Another study by Tsuchiya et al. with a similar cryosurgery technique showed excellent limb function in most subjects and a high bony union rate [21]. In this case, we found radiological union in 6 months followup with the musculoskeletal society tumor score of 76,6%. However, complications were reported in 25% of the subjects with infection (10,5%), fracture (7,5%), and local recurrence (7,5%) [16], [21]. Despite the complications mentioned, cryosurgery can serve as one of the option for biological reconstruction in osteosarcoma [23], [27]

## 4 Conclusion

Cryosurgery and vascularized fibular graft is a viable reconstructive option for proximal tibia osteosarcoma in very young children.

## Availability of data and material

None.

## Code availability

None.

## Provenance and peer review

Not commissioned, externally peer-reviewed.

## Funding

This research did not receive any specific grant from funding agencies in the public, commercial, or not-for-profit sectors.

## Ethical Approval

Ethical approval was not required in the treatment of the patient in this report.

## Consent

Written informed consent was obtained from her parents and the patient gave assent for publication of this case report and accompanying images. A copy of the written consent is available for review by the Editor-in-Chief of this journal on request.

## Author contribution

Sigit Daru Cahyadi contributes in the study concept or design, data collection, analysis and interpretation, oversight and leadership responsibility for the research activity planning and execution, including mentorship external to the core team.

Uno Surgery Erwin contributes to the study concept or design, data collection and writing the paper.

## Registration of research studies

Does not need any registration.

## Guarantor

Sigit Daru Cahyadi, MD.

## Declaration of competing interest

The authors declare no conflicts of interest.
